# 4159 Prostate cancer multiparametric MRI comparison study of 3T versus 7T in terms of lesion detection and image quality

**DOI:** 10.1017/cts.2020.141

**Published:** 2020-07-29

**Authors:** Ethan Leng, Benjamin Spilseth, Anil Chauhan, Joseph Gill, Ana Rosa, Joseph Koopmeiners, Christopher Warlick, Gregory Metzger

**Affiliations:** 1 University of Minnesota CTSI

## Abstract

OBJECTIVES/GOALS: The goal of this study was to perform a comparative, multi-reader, retrospective clinical evaluation of prostate multiparametric MRI (mpMRI) at 3 Tesla (3T) vs. 7 Tesla (7T) primarily in terms of prostate cancer localization. Subjective measures of image quality and artifacts were also evaluated. METHODS/STUDY POPULATION: Nineteen subjects were imaged at 3T and 7T between March 2016 and October 2018 under IRB-approved protocols. Four radiologists retrospectively and independently reviewed the data, and completed a two-part assessment for each dataset. First, readers assessed likelihood of cancer using Prostate Imaging Reporting & Data System (PI-RADS) guidelines. Accuracy of cancer detection was compared to findings from prostate biopsy. The numbers of correctly or incorrectly classified sextants were summed across all four readers, then used to summarize detection performance. Second, readers assigned a score on a five-point Likert scale to multiple image quality characteristics for the 3T and 7T datasets. RESULTS/ANTICIPATED RESULTS: Sensitivity and specificity of 3T and 7T datasets for sextant-wise cancer detection were compared by paired two-tailed t-tests. Readers identified more sextants harboring cancer with the 3T datasets while false-positive rates were similar, resulting in significantly higher sensitivity at 3T with no significant differences in specificity. Likert scores for image quality characteristics for 3T and 7T datasets were compared by applying paired two-tailed t-tests to mean scores of the four radiologists for each dataset. Readers generally preferred the 3T datasets, in particular for staging and assessment of extraprostatic extension as well as overall quality of the contrast-enhanced data. DISCUSSION/SIGNIFICANCE OF IMPACT: Readers agreed 7T prostate mpMRI produced images with more anatomic detail, though with equivocal clinical relevance and more pronounced artifacts. Reader unfamiliarity with 7T images is a major extenuating factor. Forthcoming technological developments are anticipated to improve upon the results.

